# Solvato/Vapochromism‐Based Alcohol Sensing through Metal–Organic Framework Thin Films with Coordinatively Unsaturated Metal Sites

**DOI:** 10.1002/smsc.202400634

**Published:** 2025-02-22

**Authors:** Yuto Toki, Kenji Okada, Arisa Fukatsu, Yuta Tsuji, Masahide Takahashi

**Affiliations:** ^1^ Department of Materials Science Graduate School of Engineering Osaka Metropolitan University 1‐1 Gakuen‐cho, Naka‐ku Sakai Osaka 599‐8531 Japan; ^2^ Faculty of Engineering Sciences Kyushu University 6‐1, Kasuga‐koen, Kasuga Fukuoka 816‐8580 Japan

**Keywords:** colorimetric sensors, metal–organic frameworks, porous functional materials, smartphone‐based sensors, solvatochromism, thin films, vapochromism

## Abstract

Ethanol (EtOH) is a ubiquitous compound with critical applications across various industries, necessitating accurate and reliable sensing for sanitation, quality control, and environmental monitoring. Chromism‐based sensors, known for their simplicity, portability, and real‐time detection capabilities, have faced limitations in EtOH sensing due to insufficient sensitivity, low selectivity, irreversibility, and low color perception. Herein, a groundbreaking solvato/vapochromism‐based EtOH sensor utilizing a Cu‐based metal–organic framework (MOF) thin film, Cu‐MOF‐74, is reported. The conversion of Cu‐based ceramics to Cu‐MOF‐74 facilitates the fabrication of solvato/vapochromic MOF thin films with low light scattering, enabling effective colorimetric analysis. The Cu‐MOF‐74 thin films demonstrate rapid and reversible solvato/vapochromism upon the adsorption of guest molecules, including water and EtOH. This unique behavior allows for the precise and reliable detection of EtOH across the entire concentration range. Furthermore, a smartphone application is developed to detect EtOH concentrations, enabling rapid and convenient evaluation of EtOH levels. The findings represent a significant advancement in EtOH sensing technology, overcoming the limitations of traditional methods. The Cu‐MOF‐74‐based sensor offers a versatile and reliable solution for various applications, including environmental monitoring, process control, and healthcare.

## Graphical abstract

Cu‐based metal–organic framework (MOF) thin film, Cu‐MOF‐74, is fabricated and exhibits rapid and reversible solvato/vapochromism upon gas adsorption, including water and ethanol. A smartphone application is developed to detect ethanol concentrations, enabling convenient evaluation of ethanol level of the surrounding atmosphere. Cu‐MOF‐74‐based sensors offer a reliable solution for various applications, including environmental monitoring, process control, and healthcare.

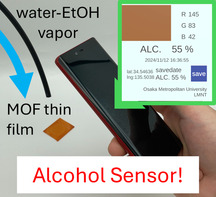

## 1 Introduction

Ethanol (EtOH) is one of the most widely used substances across various industries, including pharmaceuticals,^[^ ^1^ ^]^ foods,^[^ ^2^ ^]^ and fuels,^[^ ^3^ ^]^ as well as in laboratory studies.^[^ ^4^ ^]^ For instance, water‐EtOH mixtures with a broad range of EtOH concentrations are present in fermented foods and alcoholic beverages; thus, determining the concentration of EtOH in the presence of water is crucial for controlling the production process and monitoring and maintaining product quality.^[^ ^5^ ^]^ Semiconductor‐type gas sensors using metal oxides such as SnO_2_, Cu_2_O, and ZnO have been extensively investigated and are commercially available for determining their concentrations due to their small size and low power consumption.^[^ ^6^, ^7^ ^]^ However, these types of gas sensors require external electric power sources to measure changes in resistance upon the adsorption of target molecules. One of the simplest and most environmentally friendly gas sensors is colorimetric sensing, which utilizes solvato/vapochromism.^[^ ^8^ ^]^ Nevertheless, to the best of our knowledge, there have been few reports on vapochromic‐type EtOH sensors, largely due to the scarcity of materials that exhibit vapochromic properties while also demonstrating high surface area and selective adsorption of target gas molecules. Therefore, advanced vapochromic materials are desirable for eco‐friendly EtOH sensors with high sensitivity for colorimetric‐type gas sensing.

Metal–organic frameworks (MOFs), which are porous materials formed by the regular combination of metal ions and organic ligands, are promising candidates for high‐sensitivity sensors due to their ultrahigh porosity, ordered pore size, and tunable chemical and physical properties.^[^ ^9^, ^10^, ^11^, ^12^, ^13^ ^]^ For instance, the MOF{[(WS_4_Cu_4_)I_2_(dptz)_3_]·DMF}_*n*_ (dptz: 3,6‐di(pyridine‐4‐yl)‐1,2,4,5‐tetrazine, DMF: *N,N*‐dimethylformamide) demonstrated significant potential as a chromism‐type sensor with high sensitivity and selectivity, as its color changes upon the adsorption of molecules such as water, methanol, and chloroform. This change is attributed to modifications in the electronic structure of the MOF due to interactions between the framework and the adsorbed molecules.^[^ ^14^ ^]^ For vapochromism‐type EtOH sensors, the Co_3_[Co(CN)_6_]_2_ MOF has been reported to exhibit a color change from pink to blue depending on the concentration of EtOH in water. This occurs because the coordination geometry of Co^2+^ changes from octahedral to tetrahedral upon the substitution of the adsorbed molecule from water to EtOH.^[^ ^15^ ^]^ However, the detection of EtOH concentration is limited to ≈60% or less in Co_3_[Co(CN)_6_]_2_, probably because the binding energy of EtOH to Co^2+^ sites is higher than that of water to Co^2+^. When the concentration of EtOH exceeds ≈60%, only EtOH is adsorbed in the MOF pores. Thus, the development of a suitable MOF that exhibits vapochromism across a wide range of EtOH concentrations in water will facilitate the advancement of MOF‐based EtOH sensors.

M‐MOF‐74, also known as CPO‐27‐M, M_2_(dobdc) (M = Mg, Ti, V, Mn, etc., dobdc = 2,5‐dioxido‐1,4‐benzendicarboxylate) features one‐dimensional pores ≈1.1 nm in diameter and open‐metal sites within the pores (Figure S1, Supporting Information).^[^ ^16^, ^17^ ^]^ The open metal sites in the pores of M‐MOF‐74 afforded strong interactions with gas molecules, making them suitable for gas adsorption^[^ ^18^, ^19^ ^]^ and catalysis.^[^ ^20^, ^21^ ^]^ As vapochromism can occur due to changes in the coordination geometry of transition metals upon gas adsorption, M‐MOF‐74 is well‐suited for vapochrom‐based sensors. Caro et al. reported that in the UV–vis spectra of Co‐MOF‐74, the peak position resulting from the *d*‐*d* transition of Co shifted with the adsorption of molecules, attributed to different interaction energies between the open metal site and the adsorbed molecules.^[^ ^22^ ^]^ However, the strong interactions between the open metal sites and the adsorbed molecules lead to high energy costs for sensor reactivation (desorption of adsorbed molecules), which may involve high temperatures, reduced pressures, and/or significant time investments, such as several days of immersion in a solvent.^[^ ^23^, ^24^ ^]^ Among the M‐MOF‐74 series, theoretical studies indicate that Cu‐MOF‐74 exhibits the lowest binding energy between the open metal site and the adsorbed molecules due to the distortion of the Cu^2+^ coordination geometry and electrons occupying the antibonding orbitals;^[^ ^25^ ^]^ thus, the adsorbed molecules can be easily desorbed or exchanged with other gas molecules. In fact, Cu‐MOF‐74 demonstrated gradual desorption of water molecules at relatively low temperatures (<340 K) under a flowing inert gas without requiring substantial structural change energies,^[^ ^26^ ^]^ suggesting that both water and other gas molecules, such as EtOH, could be coadsorbed in Cu‐MOF‐74 under mixed vapors. Furthermore, Cu‐MOF‐74 exhibits vapochromism because Cu possesses unsaturated *d* orbitals, similar to those of Co. Therefore, Cu‐MOF‐74 is a promising MOF for chromism sensor detection of EtOH in water (both vapor and liquid). However, the chromism of Cu‐MOF‐74 has not been reported, likely because Cu‐MOF‐74 in its conventional powder form results in significant light losses due to scattering from its rough surface.^[^ ^23^, ^24^ ^]^ Detection of color changes in colorimetric sensors is preferable using transparent thin‐film forms with low light scattering, which are advantageous for device applications. However, conventional protocols using homogeneous solutions or suspensions are rarely employed to fabricate thin‐film Cu‐MOF‐74 owing to the production of insoluble microcrystals.^[^ ^23^, ^24^, ^27^ ^]^

This study is the first to report a MOF‐based EtOH sensor capable of full‐range detection through the solvato/vapochromism of a Cu‐MOF‐74 thin film. A method for converting ceramics (copper hydroxide, Cu(OH)_2_) into Cu‐based MOF^[^ ^28^, ^29^ ^]^ was employed to fabricate Cu‐MOF‐74 thin films with high optical transparency. The transparent Cu‐MOF‐74 thin films exhibited solvato/vapochromism upon the adsorption of guest molecules, including water and EtOH, in both liquid and vapor phases. Theoretical studies suggest that vapochromism arises from changes in the C—O bond length of phenolate in Cu‐MOF‐74, resulting from the formation of hydrogen bonds between the adsorbed molecules and phenolate oxygen atoms. The change in the C—O bond length for each adsorbed species was confirmed using Raman spectroscopy. Furthermore, the Cu‐MOF‐74 thin films displayed vapochromism that depended on the EtOH concentration across the full range. The color change upon gas adsorption was sufficiently pronounced to be detected using a smartphone. This MOF‐based EtOH sensor is anticipated to be useful in various industries and laboratories.

## 2 Results and Discussion

### 2.1 MOF Thin Film Fabrication and Characterizations

**Figure 1:**
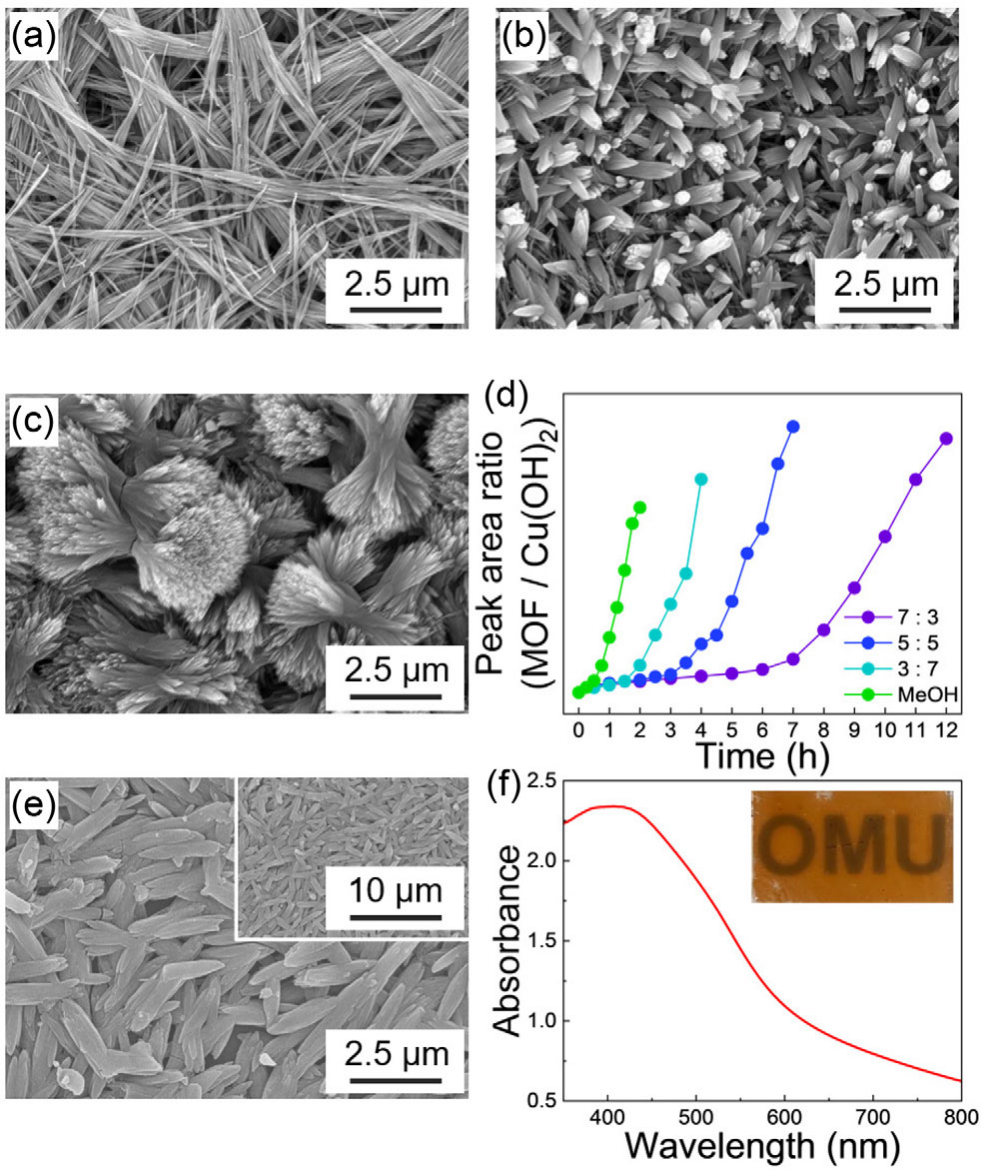
a–c) SEM images of the thin films; (a) Cu(OH)_2_ nanobelts, (b) Cu‐MOF‐74 thin film synthesized in MeOH, and (c) Cu‐MOF‐74 thin film synthesized in DMF:MeOH = 3:7. d) Peak area of the band from MOF (1556 cm^−1^) normalized to the band from Cu(OH)_2_ (3566 cm^−1^) in FT‐IR spectra with reaction time. e,f) A SEM image (e) and UV–vis absorption spectrum (f) of Cu‐MOF‐74 thin film synthesized in MeOH at 100 °C.

Cu‐MOF‐74 thin films were synthesized by a conversion method from the ceramic (Cu(OH)_2_) to Cu‐based MOF.^[^ ^28^, ^29^ ^]^ The use of Cu(OH)_2_ nanobelt assemblies as a precursor enhances the adhesion between the substrate and the converted Cu‐based MOF thin films, as well as the uniformity of the MOF crystals, as reported in previous studies.^[^ ^30^, ^31^ ^]^ Although this is the first study on the synthesis of Cu‐MOF‐74 from Cu(OH)_2_, the effect of the synthesis conditions (temperature, solvent, and reaction time) on the morphologies of the resultant MOF crystals in the thin films was investigated to achieve uniform and transparent thin films. *N,N*‐Dimethylformamide‐methanol (DMF‐MeOH) was selected as the solvent based on previous studies on the synthesis of Cu‐MOF‐74 powders from Cu salts as the metal source.^[^ ^21^, ^22^ ^]^ Scanning electron microscopy (SEM) and X‐ray diffraction (XRD) investigations revealed the formation of needle‐shaped Cu‐MOF‐74 crystals during the synthesis with MeOH as the solvent (**Figure** **1**a,b and S2, Supporting Information). In the out‐of‐plane (OOP) XRD pattern, the $23\bar{1}$ reflections were absent in the thin film synthesized using only MeOH, whereas these reflections were strongly observed in the in‐plane (IP) XRD pattern compared to the powder diffraction patterns, indicating the preferential growth of the MOF's *c*‐axis direction (representing the pore direction) along the in‐plane direction of the substrate. However, as the proportion of DMF increased in the reaction solutions, the bundles of needle‐shaped crystals with pronged edges (crystal splitting) decreased in density while their size increased (Figure 1c; S2, and S3, Supporting Information). The OOP and IP XRD patterns demonstrated that the thin film synthesized in a DMF:MeOH = 7:3 solution exhibited relatively stronger intensity for the $23\bar{1}$ reflections in the OOP direction compared to the powder diffraction patterns, indicating preferential growth of the MOF's *c*‐axis direction out of the substrate plane. Crystal splitting has been reported in various materials, including MOFs, and is caused by the slow nucleation and rapid crystal growth of the product crystals.^[^ ^32^, ^33^, ^34^ ^]^ In fact, MOF nucleation was the fastest for the MeOH solution system and slowed with increasing DMF ratio in the DMF‐MeOH mixed solution system, which was confirmed by comparing the peak area of the MOF normalized to that of Cu(OH)_2_ measured by Fourier transform infrared spectroscopy (FT‐IR) (Figure 1d and S4, Supporting Information). In the thin film synthesized by 1 h reaction time, SEM observations revealed uniform nucleation of the MOF in the MeOH (Figure S5, Supporting Information). In the DMF:MeOH = 7:3 solution, hourly SEM observations indicated that slow and nonuniform nucleation gradually began after 3 h, with rapid MOF crystal growth starting after 7 h (Figure S5, Supporting Information). These results are consistent with the findings from the FT‐IR investigation (Figure 1d). The decrease in the deprotonation rates of the organic ligands caused by mixing MeOH and DMF likely contributed to the splitting of the crystal morphology by slowing MOF nucleation, similar to a previous report.^[^ ^35^ ^]^

For chromism‐type sensors, transparent thin films with sufficient MOF‐derived light absorption and suppressed light scattering are desirable for detecting chromatic changes upon gas adsorption. Cu‐MOF‐74 thin films synthesized in solutions containing DMF were not appropriate as sensing materials because nonuniform crystal nucleation and the preferential growth of the MOF's *c*‐axis direction (longitudinal direction) perpendicular to the substrate led to many gaps that maintained the transmitted incident light and resulted in an absence of MOF‐derived light absorption (Figure S6 and S7, Supporting Information). Therefore, the Cu‐MOF‐74 thin films synthesized using MeOH as the solvent are more suitable for vapochromism‐type sensors. According to the above discussion, the reaction temperature was further optimized; synthesis in MeOH at 100 ºC resulted in the formation of a thin film that prevented light scattering by uniform crystal nucleation and growth (Figure 1e,f, S6, S7, and Table S1, Supporting Information). Therefore, the Cu‐MOF‐74 thin films synthesized at 100 °C in MeOH were used for further solvato/vapochromism experiments.

### 2.2 Vapochromic Properties of Cu‐MOF‐74 Thin Film

**Figure 2:**
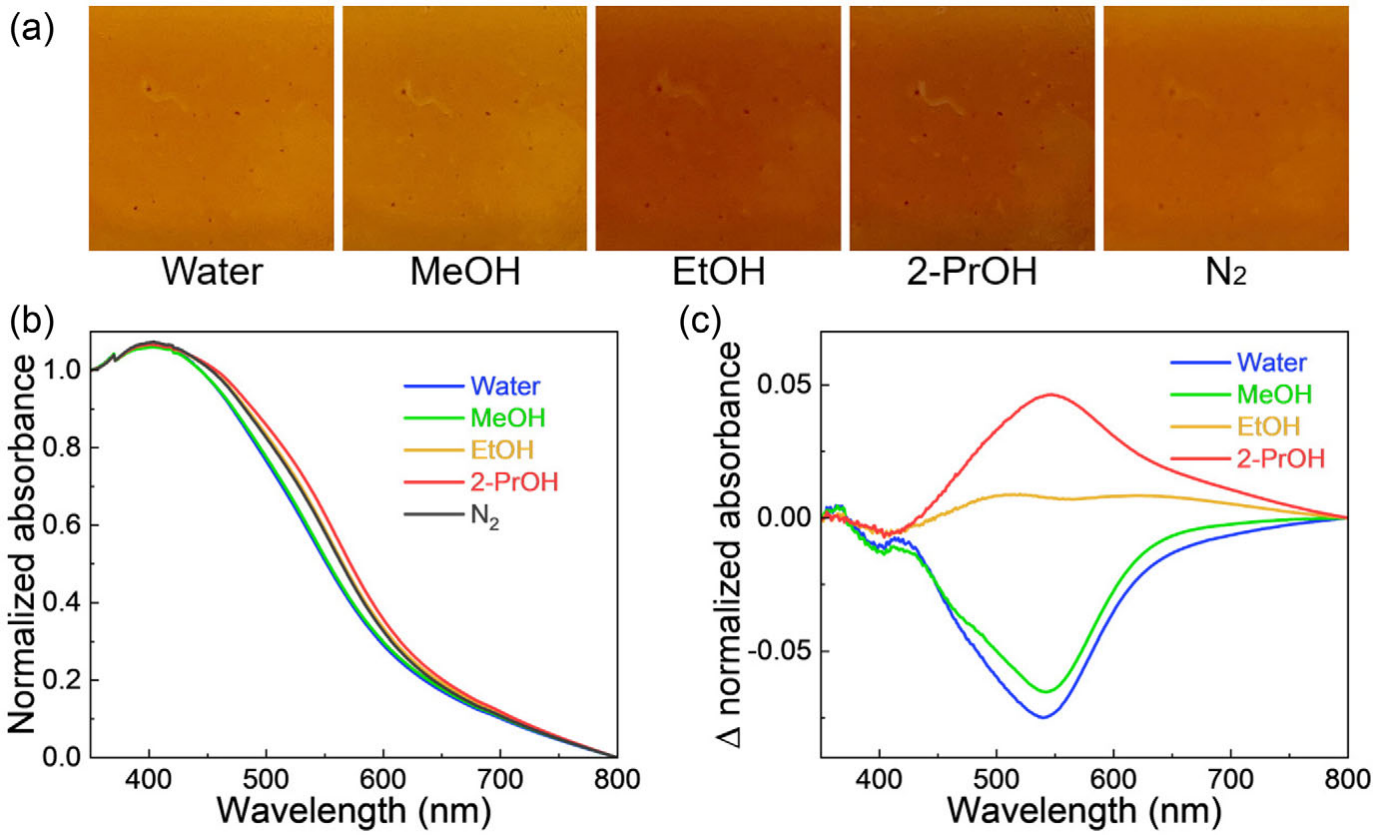
a) Photographs of the Cu‐MOF‐74 thin films under each gas or vapor. b,c) Normalized absorption spectra of the MOF thin film under N_2_ gas or different vapors atmosphere (b) and difference spectra: the absorption spectra measured under different vapors atmosphere were subtracted by that measured under N_2_ atmosphere (c).

The Cu‐MOF‐74 thin films exposed to a different type of vapor exhibited changes in color and UV–vis spectra depending on the adsorbed molecules (**Figure** **2** and S8, Supporting Information). The UV–vis difference spectra before and after vapor absorption clearly indicate that vapochromism depends on the type of absorbent used. Under water or MeOH vapor, the absorbance at 540 nm decreased compared to that in an N_2_ atmosphere. In contrast, in the presence of EtOH or 2‐propanol (2‐PrOH) vapor, the absorbance at 540 nm gradually increased with an increase in the number of alkyl chains. These color changes upon vapor adsorption were restored by N_2_ gas flow at room temperature (≈25 °C), and the vapochromism remained unchanged even after fifty adsorption/desorption cycles (Figure S9, Supporting Information). XRD, FT‐IR, and SEM investigations showed that the film was not damaged even after fifty cycles (Figure S10 and S11, Supporting Information). Cu‐MOF‐74 also demonstrated solvatochromism similar to vapochromism (Figure S12, Supporting Information). These results demonstrate that the Cu‐MOF‐74 thin film is a promising solvato/vapochromism‐based sensor with guest molecule selectivity.

### 2.3 Structure and Electronic Property of Cu‐MOF‐74

**Figure 3:**
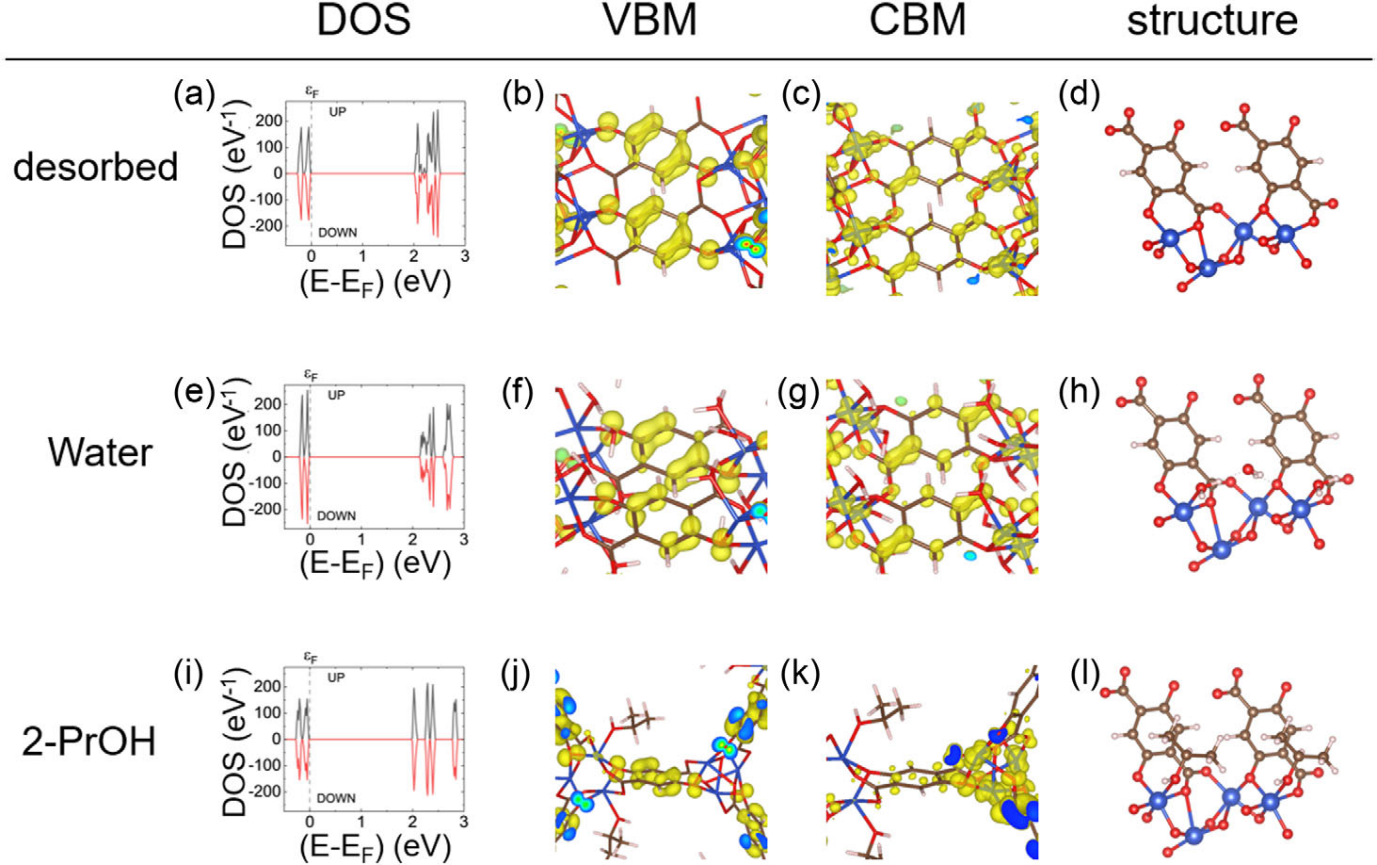
a–l) Density of states (DOS), electron density distributions for the VBM and CBM, and (a–d) structure around C—O_phenolate_ bond in the desorbed state, (e–h) the water adsorbed state, and (i–l) the 2‐PrOH adsorbed state. White, brown, red, and blue balls in the structural images represent hydrogen, carbon, oxygen, and copper, respectively.

To elucidate the chromism of Cu‐MOF‐74 upon guest adsorption, the bandgaps of Cu‐MOF‐74 thin films under different gases or vapors were estimated using a Tauc plot (**Table** **1**). The largest bandgap was observed under water vapor, decreasing as the length of the alkyl chain of the adsorbed species increased. Additionally, the bandgap was larger under water vapor and smaller under 2‐PrOH vapor than under N_2_ gas (desorption conditions). This change in the bandgap can be attributed to the strength of the interaction between the MOF and the adsorbed molecules. Indeed, in Zn‐MOF‐74, an isomorphic MOF to Cu‐MOF‐74, density functional theory (DFT) calculations demonstrated a change in the bandgap originating from the strength of the interaction between the open metal sites of the MOF and the adsorbed molecules.^[^ ^36^ ^]^ The electronic states of Cu‐MOF‐74 were calculated in the desorbed state (**Figure** **3**a‐d and S1, Supporting Information), water‐adsorbed state (Figure 3e–h and S13, S14, Supporting Information), and 2‐PrOH adsorbed state (Figure 3i–l and S13, S15, Supporting Information) using DFT calculations. Calculations for the desorbed state indicate that the orbital with an antibonding character on the C—O bonds of phenolate in the organic ligands (C—O_phenolate_) significantly contributed to the valence band maximum (VBM) of Cu‐MOF‐74. The VBM is stabilized or destabilized when the C—O_phenolate_ bond is stretched or shortened, due to the antibonding nature of the wavefunction associated with the C—O_phenolate_ bond. In contrast, the ${\text{Cu}}_{d_{x^{2}-y^{2}}}$ orbitals contributed significantly to the conduction band minimum (CBM) of Cu‐MOF‐74. The CBM is considered almost constant, regardless of the adsorbate, as the ${\text{Cu}}_{d_{x^{2}-y^{2}}}$ orbital, spreading out in the organic ligand plane, is considered to be insensitive to the adsorbate. These electronic states are similar to those in other M‐MOF‐74 series; however, in Cu‐MOF‐74, the contribution of the metal ion's orbitals is relatively larger in the CBM than that of other metal ions. This is due to the splitting of Cu^2+^ e.g., orbitals, which results in distortion of the Cu^2+^ coordination geometry.^[^ ^25^, ^37^ ^]^ Consequently, the change in the bandgap of Cu‐MOF‐74 upon guest molecule adsorption is dominated by the change in the VBM, with the alteration in the C—O_phenolate_ bond length induced by the interaction between the MOF framework and the guest molecule being the origin of the chromism in Cu‐MOF‐74. Geometric optimization revealed that the C—O_phenolate_ bond length was 0.1358 nm in the desorbed state of Cu‐MOF‐74. In the water‐adsorbed state, the C—O_phenolate_ bonds were found to be longer than that in the desorbed state, measuring 0.1360 nm, while the orbital distributions for both the VBM and CBM were almost the same as those in the desorbed state. The water molecule adsorbed on the open metal site formed a hydrogen‐bonding network, mediated by another water molecule that reached a nearby O_phenolate_ atom. This weakens the contribution of the O_phenolate_ atom to the C—O_phenolate_ bond, resulting in its elongation, which, in turn, stabilizes the VBM and increases the bandgap. In the 2‐PrOH adsorbed state, the C—O_phenolate_ bonds shortened to 0.1346 nm compared to the desorbed state. Due to steric hindrance, 2‐PrOH has difficulty forming the hydrogen‐bonded network observed in water adsorption. Therefore, in the 2‐PrOH adsorption model, hydrogen bonding, which could weaken the C—O_phenolate_ bonds, is unlikely to occur. Instead, the formation of CH—*π* interactions between the benzene ring of the ligand and the alkyl group of 2‐PrOH is suggested. The shorter C—O_phenolate_ bond destabilizes the VBM, resulting in a smaller bandgap than that of the desorbed state. In summary, small polar protic molecules cause elongation of the C—O_phenolate_ bonds due to the formation of a hydrogen bonding network between the O_phenolate_ atoms and the hydrogen atoms of the adsorbed molecules. In contrast, large polar protic molecules lead to shorter C—O_phenolate_ bonds, likely due to CH‐*π* interactions replacing hydrogen bonding. These calculated trends in C—O_phenolate_ bond lengths resulting from the adsorption of different guest molecules are supported by experimental results from in situ Raman measurements (Figure S16, Supporting Information). The peak attributed to the C—O_phenolate_ bond exhibited its lowest wavenumber under water, indicating a shortening of the C—O_phenolate_ bond. In contrast, under 2‐PrOH, the peak corresponding to the C—O_phenolate_ bond was higher than that under water and N_2_, demonstrating the elongation of the C—O_phenolate_ bond with the adsorption of 2‐PrOH. In the EtOH adsorbed state, the C–O_phenolate_ bond length is not as long as that in the water adsorbed state, nor is it as short as that in the 2‐PrOH adsorbed state. Furthermore, the band gap in the EtOH adsorbed state is smaller than that in the water adsorbed state and is close to that in the 2‐PrOH adsorbed state. These computational results coincide with the experimental results (Figure S17 and S18, Supporting Information). These data indicate that the local chemical interactions between the Cu‐MOF‐74 framework and the adsorbed molecules are the origin of the solvato/vapo‐chromism observed in Cu‐MOF‐74.

**Table 1:** Bandgaps of the MOF thin film under N_2_, water, MeOH, EtOH, and 2‐PrOH.

Flowed gas or vapor	Bandgap [eV]
N_2_	2.09
Water	2.15
MeOH	2.10
EtOH	2.05
2‐PrOH	2.03

### 2.4 Color‐Detecting Sensing of Water‐EtOH Mixed Vapor

**Figure 4:**
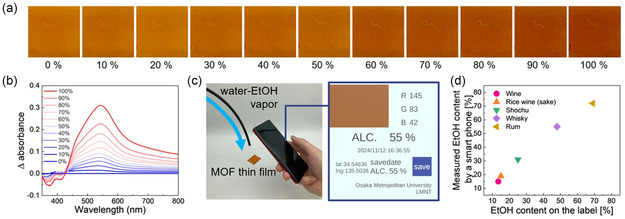
a) Photographs of the MOF thin film under water‐EtOH mixed vapor: from left to right EtOH contents increase. b) Absorption difference spectra of the MOF thin film under water‐EtOH mixed vapor. c) Demonstration of EtOH sensor by a Cu‐MOF‐74 thin film and a smartphone. d) EtOH contents in commercial alcohol beverages measured by a smartphone versus the labeled alcohol content.

Cu‐MOF‐74, characterized by its moderate desorption capacity, is anticipated to coadsorb water and EtOH from mixed vapors, reflecting the mixture ratio of both components. The color and absorption spectral changes of the Cu‐MOF‐74 thin film were investigated in water‐EtOH mixed vapors with varying EtOH concentrations (**Figure** **4**a,b). In these mixed vapors, the color of the MOF thin film gradually changed from orange to brown as the EtOH concentration in water increased, and the absorbance at 540 nm clearly depended on the EtOH concentration. This represents the first report on the full‐range detection of EtOH concentrations in MOF‐based vapochromism. The significant color and absorption spectral changes across the full range of EtOH concentrations can be attributed to Cu‐MOF‐74's high capacity for exchanging adsorbed molecules due to its moderately low binding energy for the adsorbate.

Remarkable color changes in the thin film can be analyzed using a commercially available smartphone. The RGB values of the MOF thin film, measured with a smartphone, clearly depended on the EtOH concentration (Figure S19, Supporting Information). In particular, the difference green value (Δ*G*) corresponding to a wavelength of 540 nm, which showed significant absorbance changes, exhibited the most pronounced and monotonic increase with increasing EtOH concentration. Smartphones provide a convenient analysis method because they are inexpensive, compact, portable, and user‐friendly compared to other professional spectroscopic instruments such as UV–vis spectrophotometers. Furthermore, the Cu‐MOF‐74 thin films are also small and portable. Therefore, the combined use of a Cu‐MOF‐74 thin film and a smartphone could serve as an easy‐to‐use gas sensor for detecting EtOH concentration without the need for expensive and specialized measurement devices. In fact, we demonstrated that the alcohol content of commercially available alcoholic beverages could be easily detected using the Cu‐MOF‐74 thin film and a smartphone (Figure 4c,d and S19, Supporting Information). In real‐world applications, EtOH concentrations in multicomponent environments could be measured by drawing a calibration curve using mass, FT‐IR, Raman spectroscopy, or others. Cu‐MOF‐74 thin films could be employed as EtOH gas sensors in various industries such as environmental monitoring (e.g., industrial emissions testing) and the medical field (e.g., breath alcohol analysis), particularly the alcoholic beverage industry, due to the noticeable color change observed under water‐EtOH mixed vapor across a full range of EtOH concentrations.

## 3 Conclusion

This study is the first to report an MOF‐based EtOH sensor capable of full‐range detection in water, utilizing the solvato/vapochromism of Cu‐MOF‐74 thin films. The Cu‐MOF‐74 thin films, characterized by reduced light scattering, were fabricated through uniform crystal nucleation and growth, employing a conversion method from Cu(OH)_2_ nanobelt thin films to Cu‐MOF‐74 in MeOH solvent at 100 °C. UV–vis measurements confirmed that the Cu‐MOF‐74 thin films exhibited solvato/vapochromism based on the adsorbed molecules. Theoretical studies suggest that the presence or absence of hydrogen bonding between the adsorbed molecules and phenolate oxygen atoms in Cu‐MOF‐74 leads to the elongation or shortening of the C–O_phenolate_ bond, with local chemical interactions resulting in an increase or decrease in the bandgap. The Cu‐MOF‐74 thin films displayed vapochromism depending on the EtOH concentration in water across the full range of concentrations, and the colorimetric change was analyzed using a smartphone, eliminating the need for specialized spectroscopy instruments. In fact, the EtOH concentrations in commercially available alcoholic beverages can be easily detected using Cu‐MOF‐74 thin films and smartphones. These MOF thin films exhibiting solvato/vapochromism are expected to pave the way for the development of advanced EtOH sensors with broad applications in fields such as environmental monitoring, process control, and breathalyzers.

## 4 Experimental Section

4.1

4.1.1

##### Synthesis of Cu(OH)_*2*_ *Nanobelt Suspensions*

CuSO_4_·5H_2_O, ammonia solution, and NaOH were purchased from FUJIFILM Wako Pure Chemical Corp, Osaka, Japan. Cu(OH)_2_ nanobelt suspensions were synthesized according to the previous method:^[^ ^29^ ^]^ starting from a 0.04 m CuSO_4_·5H_2_O aqueous solution (20 mL), 0.333 m NH_3_ aqueous solution (6 mL) was added dropwise under stirring, resulting in a deep blue solution. After 5 min of continuous stirring, 12 m NaOH aqueous solution (1.2 mL) was added dropwise, resulting in a light blue precipitate. The mixture was stirred for 1 h at room temperature. Subsequently, a 30 min thermal treatment at 40 °C (without stirring) was performed, and the resultant powders were centrifuged and washed with water (1 × 20 mL) and ethanol (2 × 20 mL). Then, wet Cu(OH)_2_ nanobelts were obtained (corresponding to 0.075 g of dried nanobelts) and dispersed in 10 mL ethanol.

##### Synthesis of Cu(OH)_2_ Nanobelt Thin Film on Silicon Substrate by Drop‐Cast Method

Cu(OH)_2_ nanobelt thin films were prepared on a silicon substrate by dropping the prepared Cu(OH)_2_ nanobelt suspension (100 μL) onto a 10 × 15 mm silicon substrate and allowing it to dry naturally. The amount of Cu(OH)_2_ was higher than that obtained following the spin‐coating method, and the MOF thin film synthesized from Cu(OH)_2_ nanobelt thin films obtained by drop‐casting was used for characterization by XRD, FT‐IR, SEM, and Raman spectroscopy, as described below.

##### Synthesis of Cu(OH)_2_ Nanobelt Thin Films on Glass Substrates by Spin‐Coat Method

Cu(OH)_2_ nanobelt thin films were prepared on a silica glass substrate (15 mm × 15 mm) by spin‐coating method with a rotation spread of 500 rpm. The MOF thin films synthesized from Cu(OH)_2_ nanobelt thin films obtained by spin‐coating were characterized by SEM and UV–vis spectroscopy, as described below.

##### Synthesis of Cu‐MOF‐74 Thin Films Using DMF‐MeOH Mixture Solution

Methanol and *N,N*‐dimethylformamide were purchased from FUJIFILM Wako Pure Chemical Corp, Osaka, Japan. 2,5‐dioxido‐1,4‐benzendicarboxylic acid (H_4_dobdc) was purchased from TOKYO CHEMICAL INDUSTRY CO., LTD, Japan. The Cu(OH)_2_ thin film was immersed in a MeOH or DMF‐MeOH mixture (mixing ratio was DMF:MeOH = 3:7, 5:5, 7:3) containing an organic ligand (H_4_dobdc; 2,5‐dioxido‐1,4‐benzendicarboxilic acid) (1 mm) and kept at room temperature (≈25 °C). The amount of solution used was 20 mL for the Cu(OH)_2_ thin films on the 15 mm × 15 mm silicon substrates and 5 mL for the Cu(OH)_2_ thin films on the 15 mm × 15 mm silica glass substrates. The reaction time varied from 1.5 to 48 h.

##### Synthesis of Cu‐MOF‐74 Thin Films with MeOH Solution in Different Reaction Temperatures

Cu(OH)_2_ thin film was immersed in MeOH solution containing organic ligand (1 mm), and it was kept at room temperature, 60, or 100 °C (for the reaction at 100 °C, Teflon‐lined autoclave was used). The amount of the solution used was 5 mL for the Cu(OH)_2_ thin films on 15 mm × 15 mm silica glass substrates. The reaction time was varied from 15 to 90 min.

As preliminary experiments, Cu(OH)_2_ thin films with three different numbers of the spin‐coating process were prepared for fabricating the MOF thin film with three different thicknesses to explore the effect of thickness on the sensitivity of vapochromism. The MOF thin film with 0.96 μm thickness (five times spin coating) showed the best sensitivity for vapochromism. The thinner film with 0.88 μm thickness (two times spin coating) showed lower absorption originated from Cu‐MOF‐74 and the thicker film with 2.81 μm thickness (seven times spin coating) did not show MOF‐derived absorption spectra due to light scattering. Therefore, the MOF film with 0.96 μm thickness (five times spin coating) was selected for the further vapochromism experiments.

##### Characterization

The crystal morphologies were observed using field‐emission scanning electron microscopy (FE‐SEM; SU8010; Hitachi High‐Tech Corporation, Japan). Identification and orientation evaluation of the obtained MOF crystals were performed using XRD (SmartLab; Rigaku Holdings Corporation, Japan) measurements. The conversion kinetics were investigated using FT‐IR (FT/IR‐4600ST; JASCO, Japan). The thicknesses of the thin films were determined using a microfigure measuring instrument (ET200, Kosaka Laboratory Ltd, Japan).

##### Vapochromism

Ethanol, 1‐propanol, 2‐propanol, acetone, and hexane were purchased from FUJIFILM Wako Pure Chemical Corp, Osaka, Japan. Water‐EtOH mixed vapor was supplied from water‐EtOH mixed solvent. The absorption spectra of the MOF thin films under N_2_ gas and other vapors were measured using UV–vis spectroscopy (V‐670ST; Jasco, Japan). The primary gas inlet was used to supply the vapor by regulating the gas flow (200 sccm) using a flow control system (FCS‐T1000F; Fujikin Carp Group, Japan) and passing the stream through a gas washing bottle. The secondary gas inlet used the flow control system and connected to a N_2_ tank to supply a N_2_ gas. Normalized absorption spectra represented clearly spectral shape; the absorbance at 800 nm was subtracted from zero and then the absorbance divided to 1 at 350 nm (Figure 2c, S8b, and S12, Supporting Information). Raman spectroscopy measurements (LabRamHR Evolution Vis‐NIR‐KOS; HORIBA Scientific, Japan) were conducted on the MOF thin films immersed in each solvent.

##### First Principles Calculation Method

Spin‐polarized DFT calculations were performed using the Vienna ab initio simulation package (VASP) version 5.4.4.^[^ ^38^, ^39^, ^40^ ^]^ To consider antiferromagnetic intrachain interactions and weaker ferromagnetic interchain interactions,^[^ ^41^, ^42^ ^]^ a hexagonal unit cell doubled along the *c*‐axis was constructed. The constructed unit cell contains 324 atoms. The projector‐augmented wave method^[^ ^43^ ^]^ described the interactions between ion cores and electrons. The electron exchange‐correlation was treated using the Perdew–Burke–Ernzerhof functional^[^ ^44^ ^]^ of the generalized gradient approximation with a Hubbard‐like parameter (PBE + *U*)^[^ ^45^ ^]^ to represent the nature of the 3*d* electrons of Cu. The *U*_eff_ value was set to 10 eV. This *U*_eff_ value was determined to reproduce the bandgap calculated using the Heyd–Scuseria–Ernzerhof (HSE06) hybrid functional.^[^ ^46^, ^47^ ^]^ The Tkatchenko–Scheffler method^[^ ^48^ ^]^ was used to correct for the dispersion force. The cutoff energy was set to 500 eV, and the *k*‐point mesh was set to 2π × 0.05 Å^−1^. The convergence threshold for the self‐consistent field iteration was set to 1.0 × 10^−5^ eV. The internal atomic coordinates and cell parameters were fully optimized until the forces acting on each atom were <0.03 eV Å^−1^. Optimized structures were visualized using VESTA program.^[^ ^49^ ^]^

##### Smartphone Analysis

The alcohol content analysis app was developed using Unity software,^[^ ^50^ ^]^ which allows functional app programming for smartphones. The source code of the app and more detailed information are available in the supporting information. The app was installed on a smartphone (Android 10 operating system) for analysis. First, the red, green, and blue color intensity values (RGB) of the MOF thin film under water‐EtOH mixed vapor were recorded by the smartphone; the smartphone and the MOF thin film on a white paper were placed parallel to each other at 10 cm distance and a white LED ring light was placed at 1 cm apart from the MOF thin film, and the RGB values on blank white paper were 184, 193, and 183, respectively (Figure S20, Supporting Information). The difference between the *G* value of the MOF thin film and the blank was defined as Δ*G* considered as the analytical signal, and a calculation formula (Equation S(1), Supporting Information) for calculating the EtOH concentration of vapor sources from the Δ*G* value was installed in the app. Using a smartphone installed on the app, the calculated alcohol concentrations of commercially available alcoholic beverages were recorded. Commercial alcoholic beverages used were Tanca Farra Alghero Sella & Mosca (Wine, ALC 13.5%), DASSAI39 (Rice wine (sake), ALC 15%), KAMETSUBOSHOCHU AKARUINOUSON (Shochu, ALC 25%), Ichiros Malt & Grain Classical Edition (Whisky, ALC 48%), and PLANTATION OVERPROOF O.F.T.D. (Rum, ALC 69%).

## Conflict of Interest

The authors declare no conflict of interest.

## Author Contributions

**Yuto Toki**: investigation (lead); methodology (lead); writing—original draft (lead). **Kenji Okada**: conceptualization (lead); funding acquisition (equal); project administration (equal); supervision (lead); writing—review & editing (equal). **Arisa Fukatsu**: funding acquisition (supporting); methodology (supporting); supervision (supporting); writing—original draft (supporting); writing—review & editing (supporting). **Yuta Tsuji**: funding acquisition (equal); investigation (equal); methodology (equal); writing—original draft (equal). **Masahide Takahashi**: conceptualization (equal); funding acquisition (lead); project administration (lead); supervision (lead); validation (lead); writing—review & editing (lead).

## Supporting information

Supplementary Material

## Data Availability

The data that support the findings of this study are available from the corresponding author upon reasonable request.
