# Supplementary material for: Solvato/Vapochromism‐Based Alcohol Sensing through Metal–Organic Framework Thin Films with Coordinatively Unsaturated Metal Sites
Source: Small Sci. 2025 Feb 22;5(6):2400634. doi: 10.1002/smsc.202400634 (PMC12168603; doi:10.1002/smsc.202400634)
Supplement: Supplementary file 1 — Supplementary Material [file SMSC-5-2400634-s001.zip › smsc202400634-sup-0001-SuppData-S1.pdf]

Supporting Information

Solvato/Vapochromism-Based Alcohol Sensing through Metal–Organic Framework Thin Films with Coordinatively Unsaturated Metal Sites

Yuto Toki, Kenji Okada, Arisa Fukatsu, Yuta Tsuji, Masahide Takahashi**

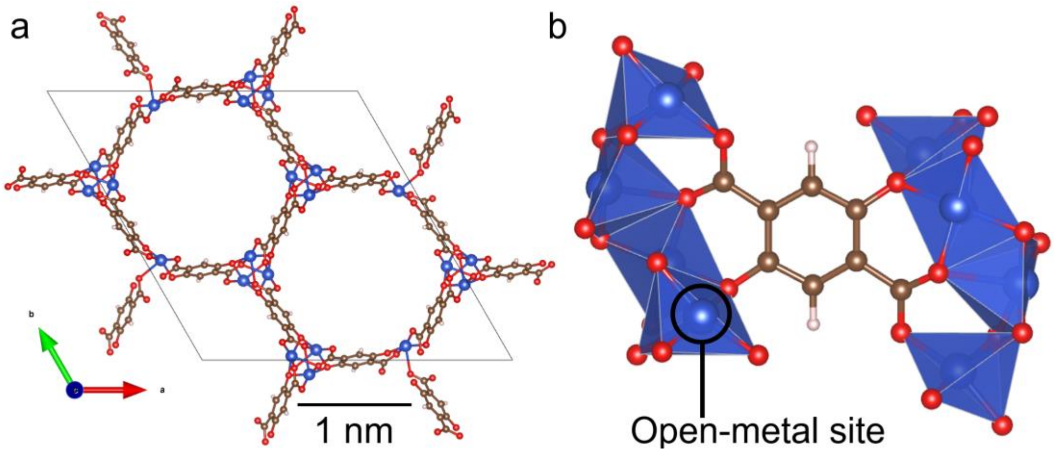

Figure S1. a) Cu-MOF-74 structural model in desorbed state. b) The configuration of the Cu-O rigid units as well as the 2,5-dioxido-1,4-benzendicarboxylate linkers; white, brown, red, and blue balls represent hydrogen, carbon, oxygen, and copper, respectively.

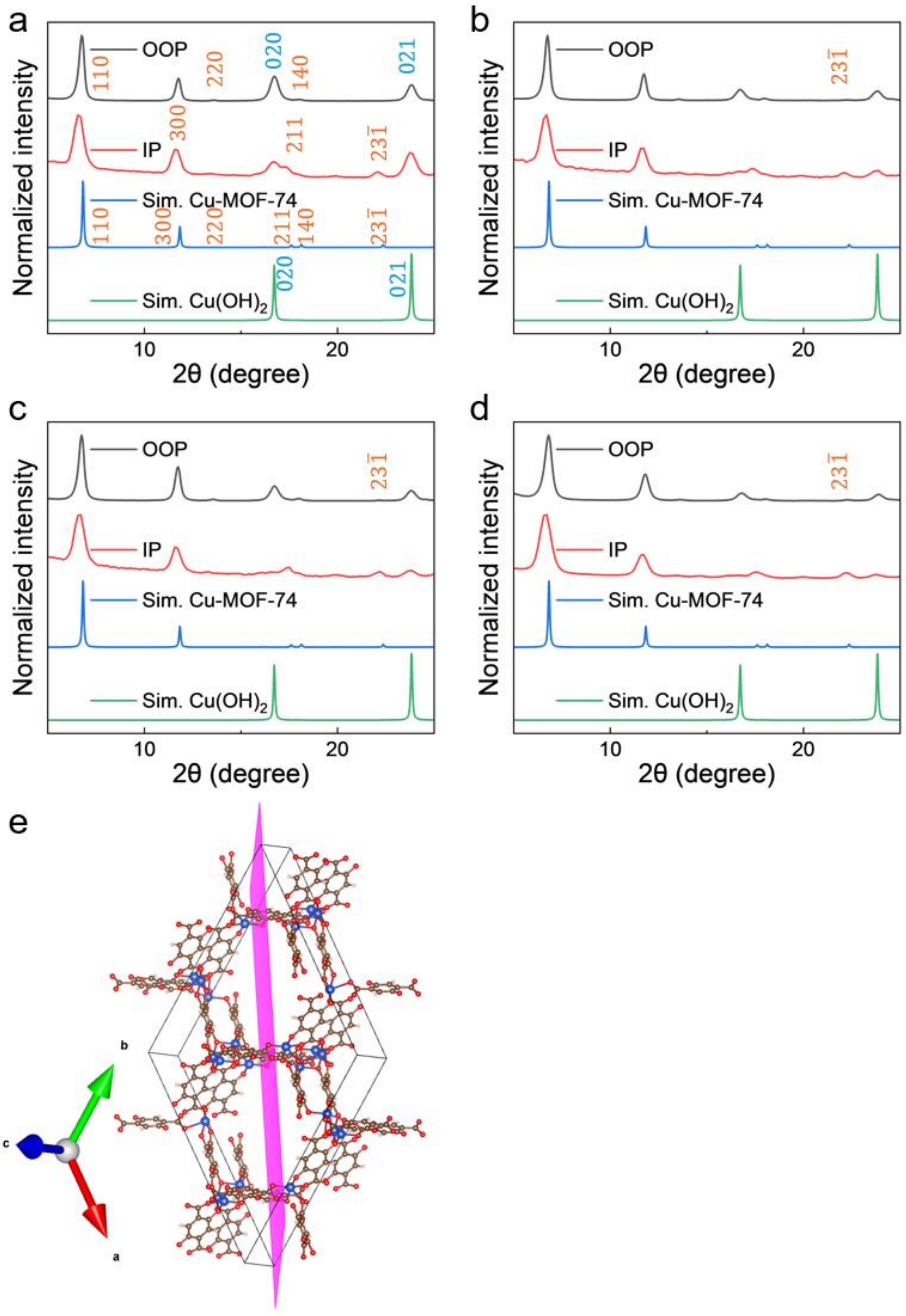

Figure S2. a-d) Out of plane (black) and in-plane (red) XRD patterns of simulated Cu-MOF-74, $Cu(OH)_2$ and the MOF thin films synthesized in MeOH (a), DMF:MeOH = 3:7 (b), 5:5 (c), and 7:3 mixture (d). Orange and light blue letters indicate the reflection indices from Cu-MOF-74 and $Cu(OH)_2$, respectively. e) Cu-MOF-74 structural model and its $23\bar{1}$ plane (pink).

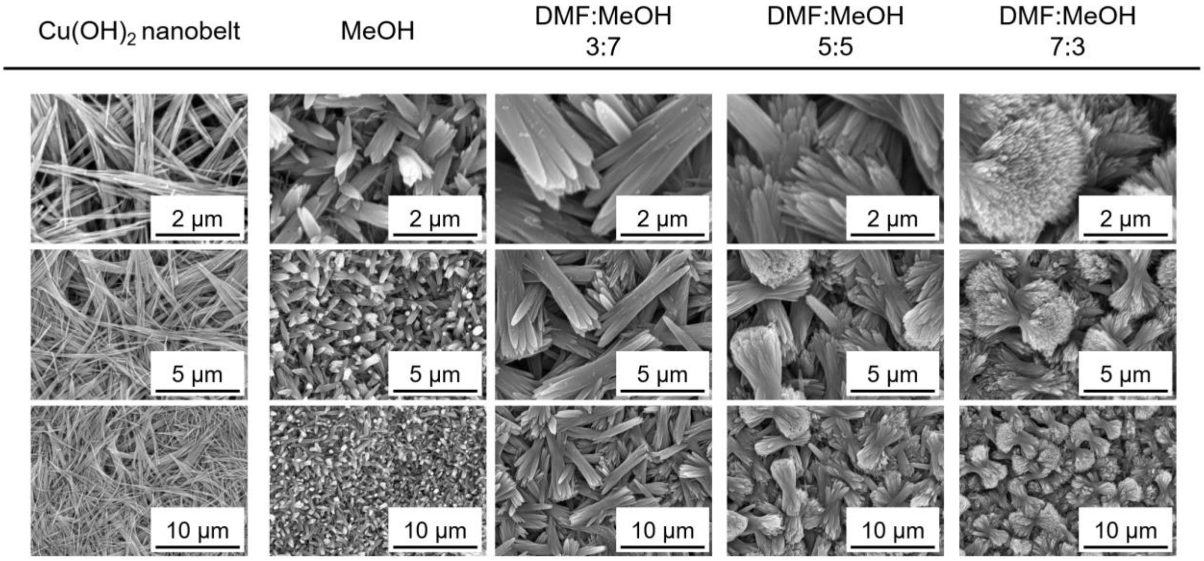

Figure S3. SEM images of the films; $Cu(OH)_2$ and MOF thin films synthesized in MeOH or DMF-MeOH mixture of different mixing ratio.

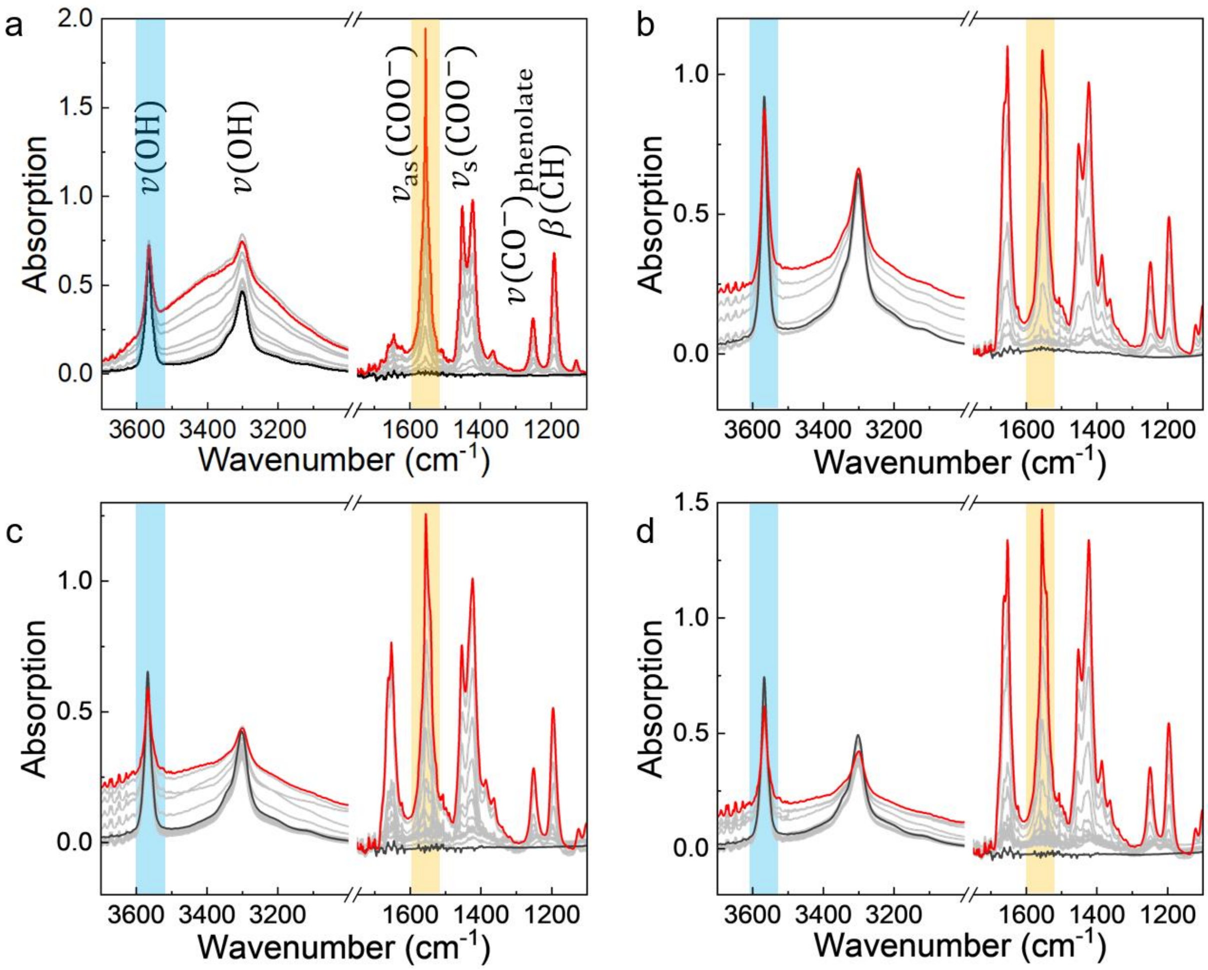

Figure S4. a-d) FT-IR spectra of MOF thin films synthesized in MeOH (a), DMF:MeOH = 3:7 (b), 5:5 (c), and 7:3 mixture (d).[1] Orange and light blue highlighted areas show the bands of MOF and $Cu(OH)_2$, respectively. The bands were used for conversion kinetics investigations.

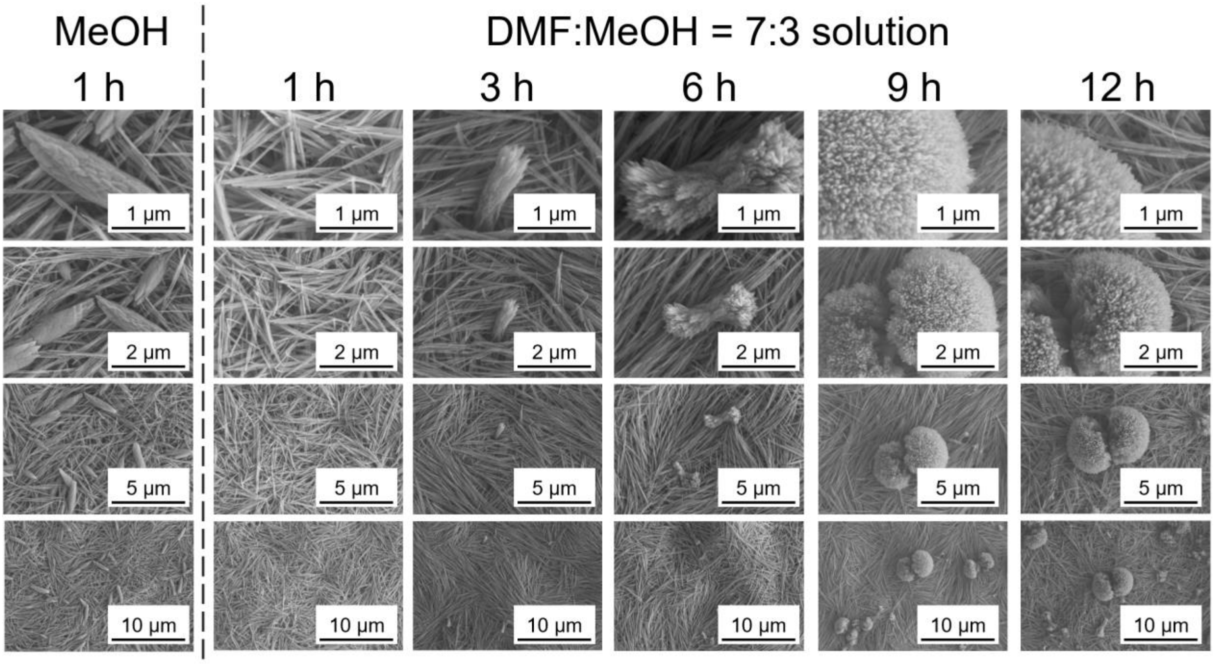

Figure S5. SEM images of the MOF thin films synthesized in MeOH or DMF:MeOH = 7:3 mixture with different reaction time.

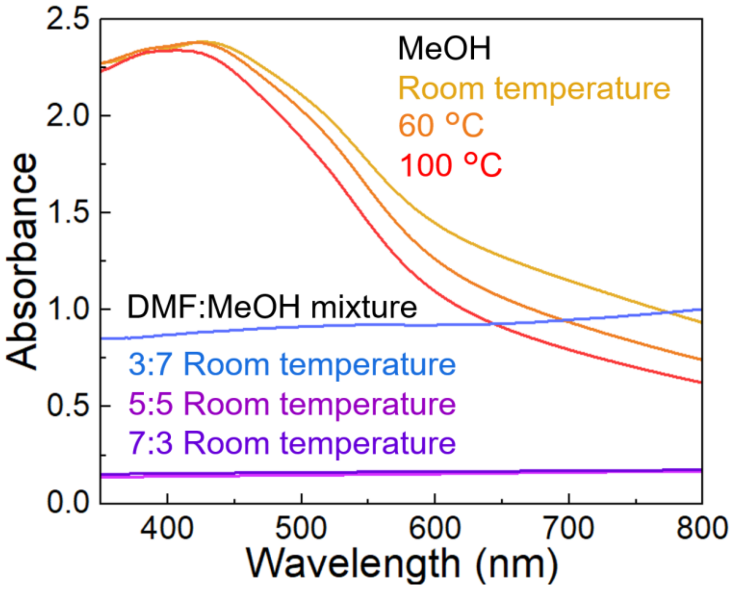

Figure S6. UV-vis absorption spectra of the MOF thin films synthesized in MeOH or DMF-MeOH mixture at different temperature.

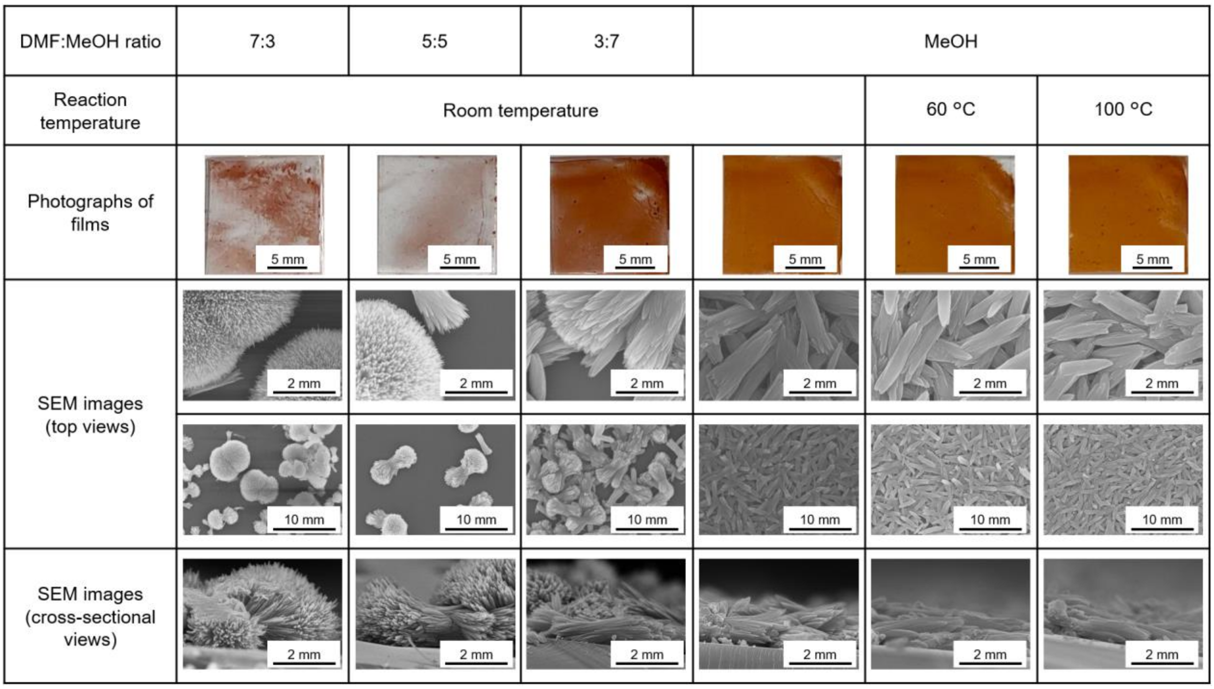

Figure S7. SEM and photo images of the MOF thin films synthesized in MeOH or DMF-MeOH mixed solution at different temperatures.

Table S1. Thicknesses of the MOF thin films synthesized in the MeOH under different temperatures.

Reaction temperature	Thickness [μm]
Room temperature (~25 °C)	1.16
60 °C	0.96
100 °C	0.93

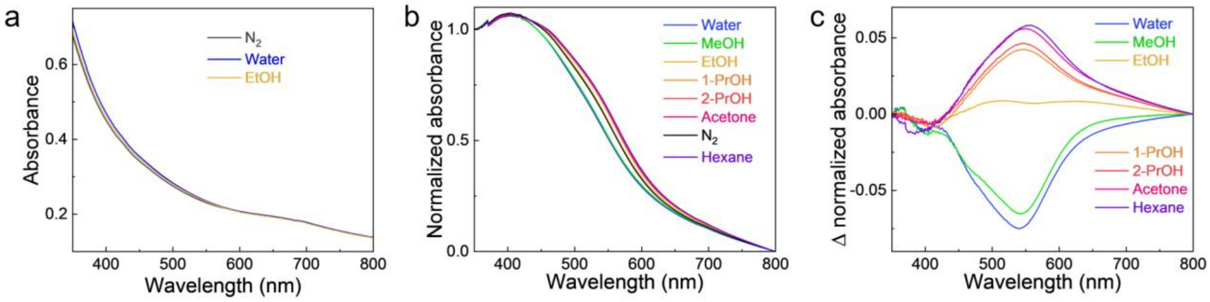

Figure S8. a) Absorption spectra of the $Cu(OH)_2$ nanobelts under N_2 gas or different vapors atmosphere. b-c) Normalized absorption spectra of the MOF thin film under N_2 gas or different vapors atmosphere (b) and difference spectra: the absorption spectra measured under different vapors atmosphere were subtracted by that measured under N_2 atmosphere (c).

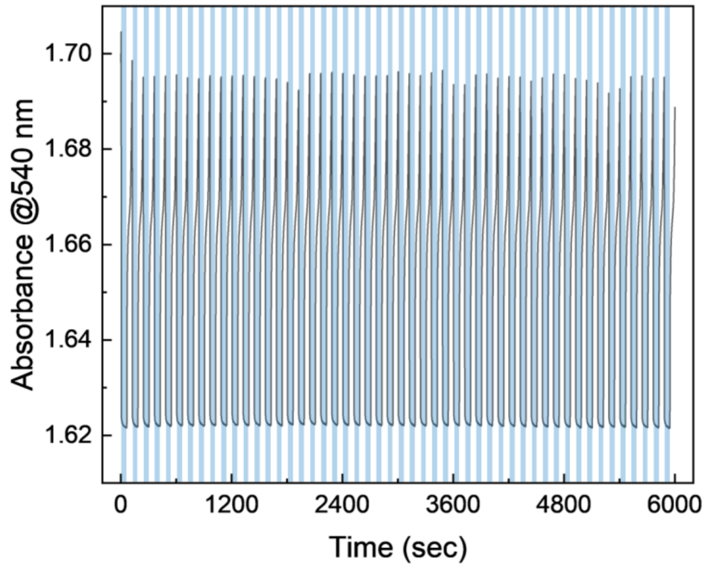

Figure S9. Absorbance at 540 nm measured under alternating flow of N_2 gas and water vapor (blue highlighted area).

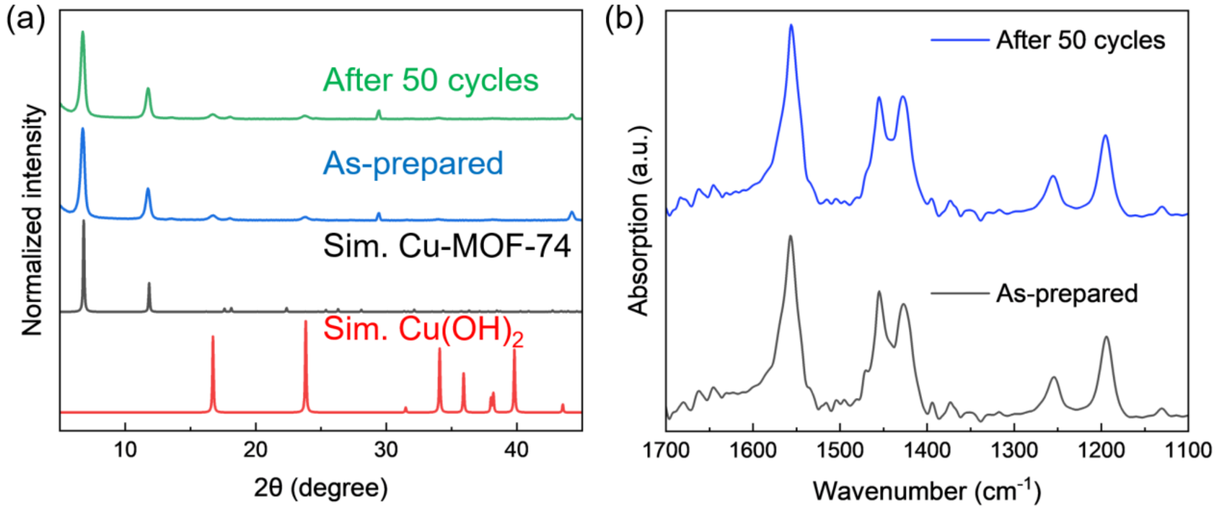

Figure S10. a) XRD patterns and (b) FT-IR spectra of as-prepared MOF thin film and the MOF thin film after 50 adsorption/desorption cycles.

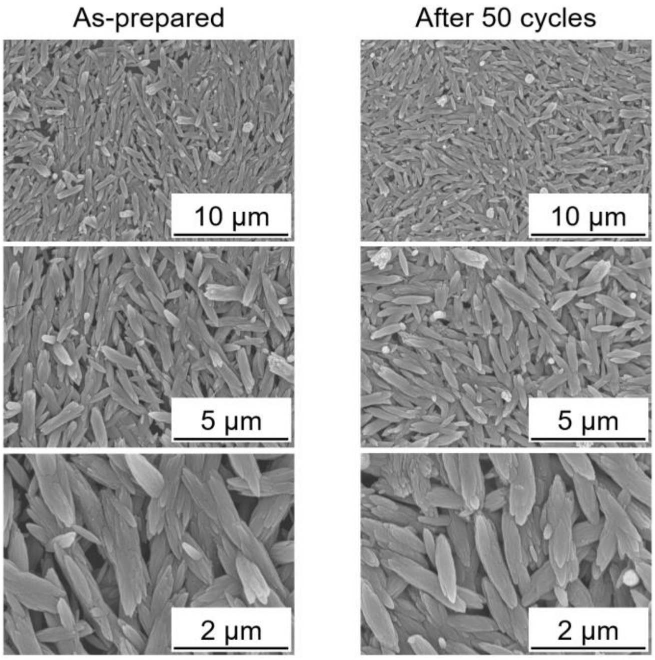

Figure S11. SEM images of as-prepared MOF thin film and the MOF thin film after 50 adsorption/desorption cycles.

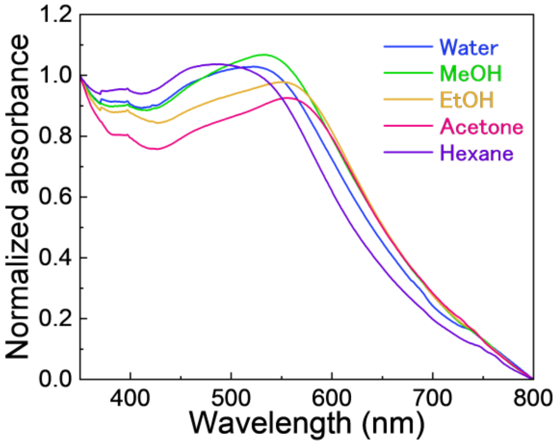

Figure S12. Normalized absorption spectra of the MOF thin films immersed in different solvents (solvatochromism).

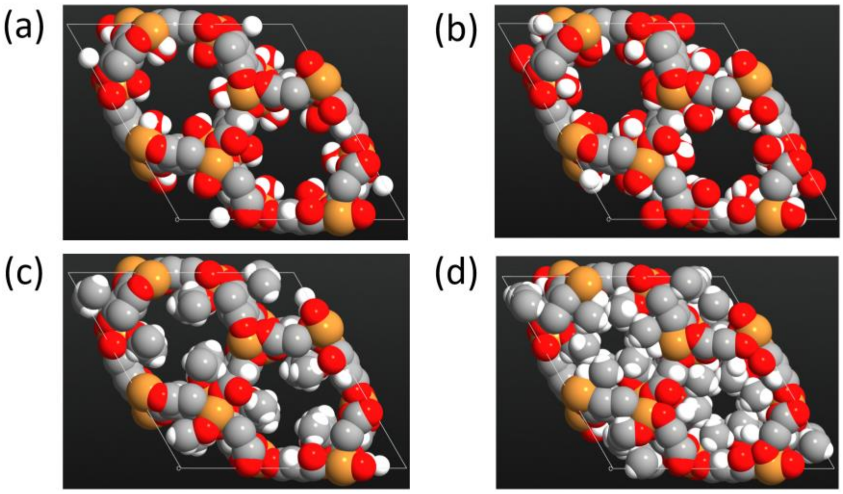

Figure S13. a-d) van der Waals representations of the structures of Cu-MOF-74 (a) with one water molecule adsorbed at each open metal site, (b) with one water molecule adsorbed at each open metal site and an additional water molecule hydrogen-bonded to it, (c) with 2-PrOH adsorbed at half of the open metal sites, and (d) with one 2-PrOH molecule adsorbed at each open metal site. These structures were visualized using the QuantumATK NanoLab software package[2].

In the case of 2-PrOH adsorption, it is likely that due to spatial constraints within the channel, the coverage will only reach a level where approximately half of the open metal sites are occupied. On the other hand, for water adsorption, even when all the open metal sites are occupied by water, there is still sufficient space to accommodate additional water molecules. As a result, water molecules adsorbed on the open metal sites can interact with additional water molecules through hydrogen bonding. This assumption is supported by visualizations of the adsorption structures using van der Waals representations, while varying the coverage of 2-PrOH and water molecules (Figure S13).

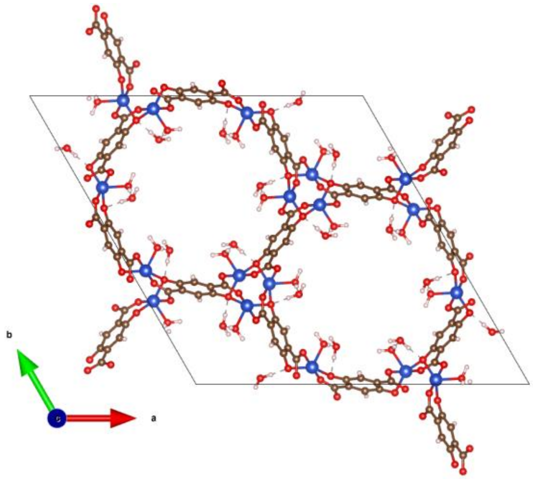

Figure S14. Cu-MOF-74 structural model in water adsorbed state; white, brown, red, and blue balls represent hydrogen, carbon, oxygen, and copper, respectively.

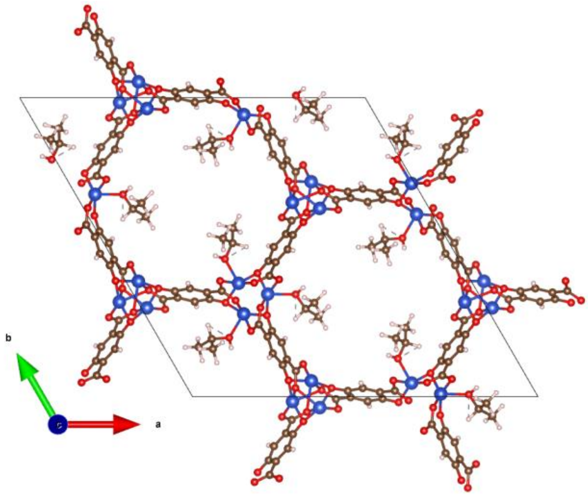

Figure S15. Cu-MOF-74 structural model in 2-PrOH adsorbed state; white, brown, red, and blue balls represent hydrogen, carbon, oxygen, and copper, respectively.

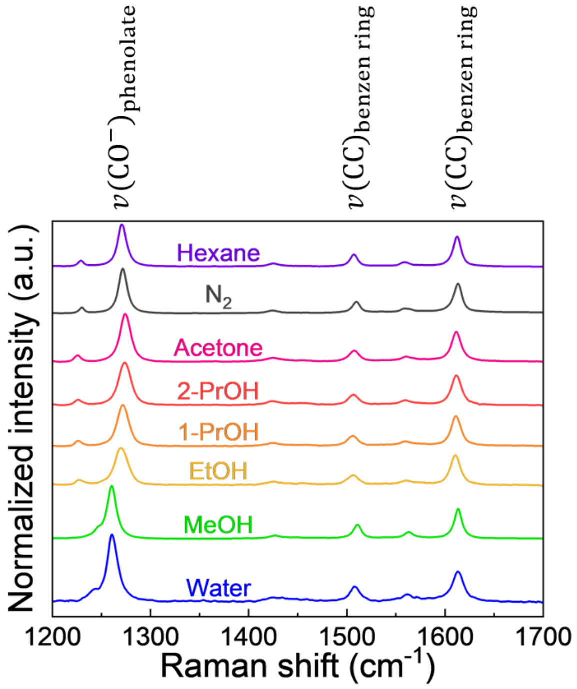

Figure S16. Raman spectra of the MOF thin film under N_2 gas or in different solvents[1, 3]

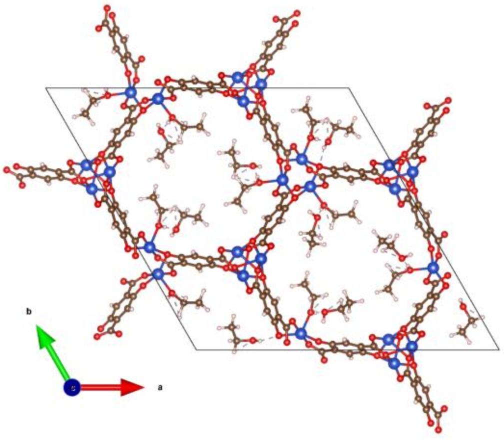

Figure S17. Cu-MOF-74 structural model in EtOH adsorbed state; white, brown, red, and blue balls represent hydrogen, carbon, oxygen, and copper, respectively.

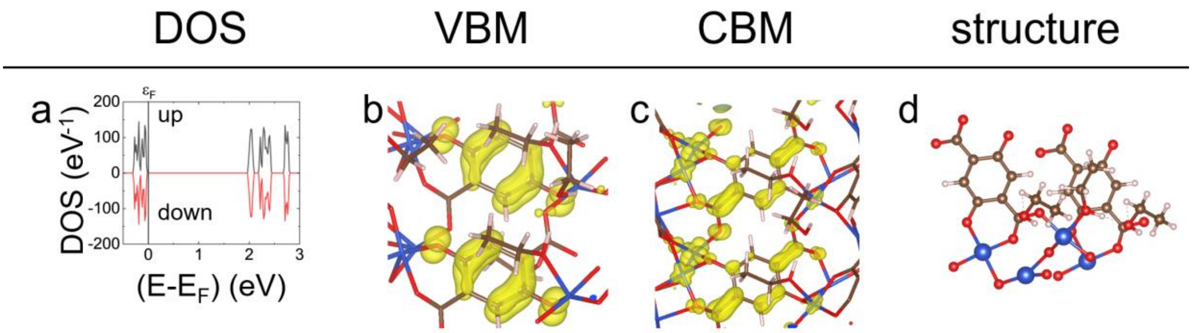

Figure S18. a-d) DOS, electron density distributions for the VBM and CBM, and structure around C-$O_{phenolate}$ bond in the EtOH adsorbed state.

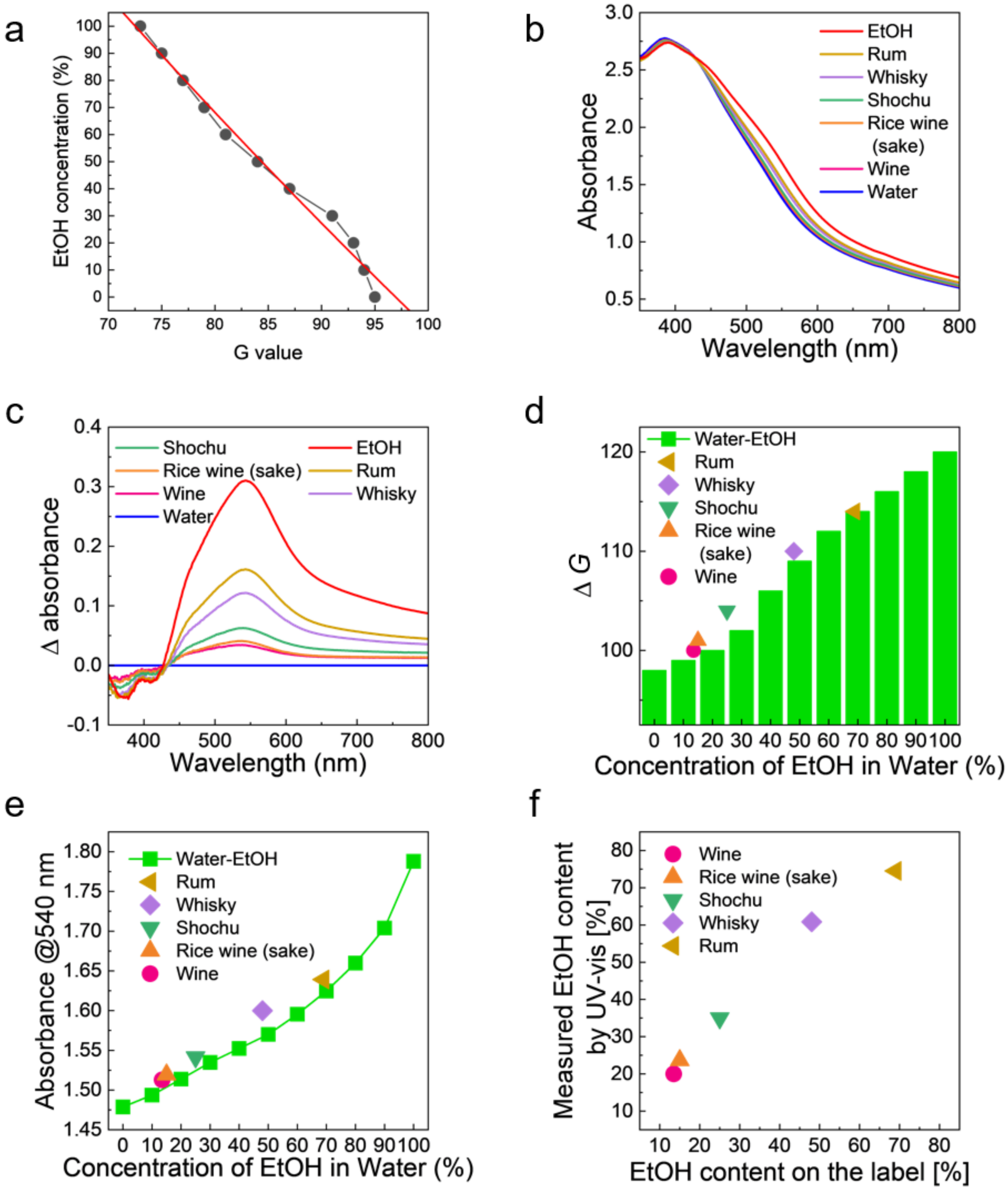

Figure S19. a) Relation between the *G* value of the MOF thin film and the EtOH concentration of water-EtOH mixed vapors (black dots) (Red line indicates the approximate curve). b-c) Absorption spectra of the MOF thin film under water, EtOH, or commercial alcohol beverages' vapors (b) and their difference spectra to water (c). d) Δ*G* value of the MOF thin film under water-EtOH mixed vapors and commercial alcohol beverages' vapors. e) Absorbance at 540 nm of the MOF thin film under water-EtOH mixed vapors and commercial alcohol beverages' vapors. f) EtOH contents of commercial alcohol beverages measured by UV-vis versus that on the labels.

$$y = 0.0097x^2 - 5.7394x + 465.24 \quad \text{(S1)}$$

Equation S1 was calculated from *G* values of the MOF thin film under water-EtOH mixed vapor, where *y* and *x* represent the EtOH concentration and the *G* value, respectively (Figure S19 a). Its coefficient of determination is 0.986.

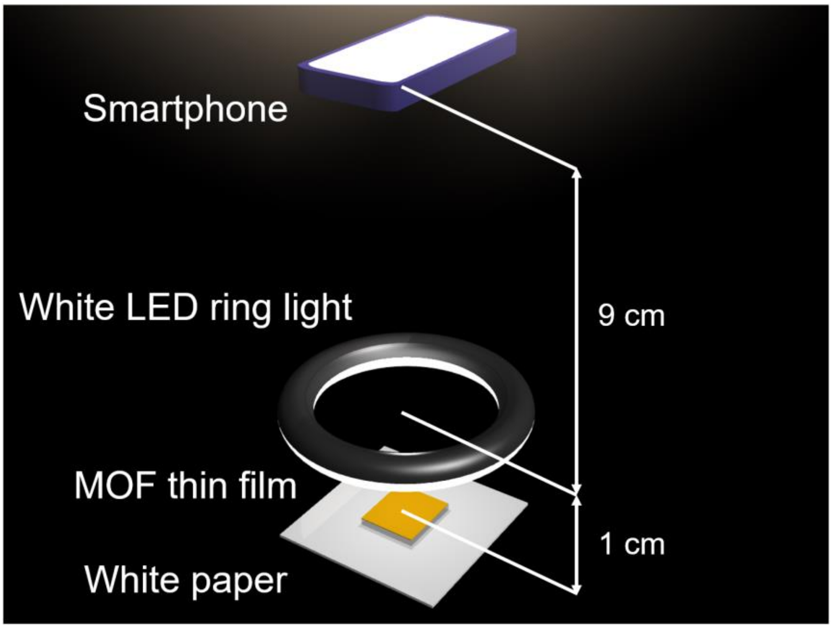

Figure S20. Illustration showing the configuration of the smartphone analysis.

Table S2. Discussion of the reported EtOH sensing techniques.

Principle	Detection range of EtOH	Reference
Vapochromism	0-100 %	This work
Vapochromism	0-60 %	4
Vapochromism	Detection from dilute concentrations	5
Solvatochromism	0-5 %	6
Photonic crystal	10-70 %	7
Fluorescent	0-100 %	8
Titration	0-50 %	9

Reference

[1] N. Drenchev, M. H. Rosnes, P. D. C. Dietzel, A. Albinati, K. Hadjiivanov, P. A. Georgiev, *J.Phys. Chem. C,* **2018**, *122*, 17238-17249.

[2] QuantumATK version S-2021.06. in Synopsys QuantumATK 2021. https://www.synopsys.com/silicon/quantumatk.html, 10, **2024**

[3] S. M. Angel, N. S. Barnett, A. A. Talin, M. E. Foster, V. Stavila, M. D. Allendorf, M. C. So, *J. Mater. Chem. C*, **2024**, *12*, 2699.

[4] Z. Wang, Q. Chen, *Spectrochim Acta A Mol Biomol Spectrosc* **2018**, *194*, 158.

[5] E. Li, K. Jie, Y. Zhou, R. Zhao, B. Zhang, Q. Wang, J. Liu, F. Huang, A*CS Appl. Mater. Interfaces* **2018**, *10*, 27, 23147–23153.

[6] A. Shahvar, D. Shamsaei, M. Saraji, *Measurement*, **2020**, *150,* 107068.

[7] M. Heshmat, P. C. H. Li, *ACS Omega,* **2019**, *4,* 22, 19991–19999.

[8] R. Huang, K. Liu, H. Liu, G. Wang, T. Liu, R. Miao, H. Peng, Yu. Fang, *Anal. Chem.*, **2018**, *90*, 23, 14088–14093.

[9] S. A. Nogueira, A. D. Lemes, A. C. Chagas, M. L. Vieira, M. Talhavini, P. A.O. Morais, W. K.T. Coltro, *Talanta*, **2019**, *194*, 363-369.
